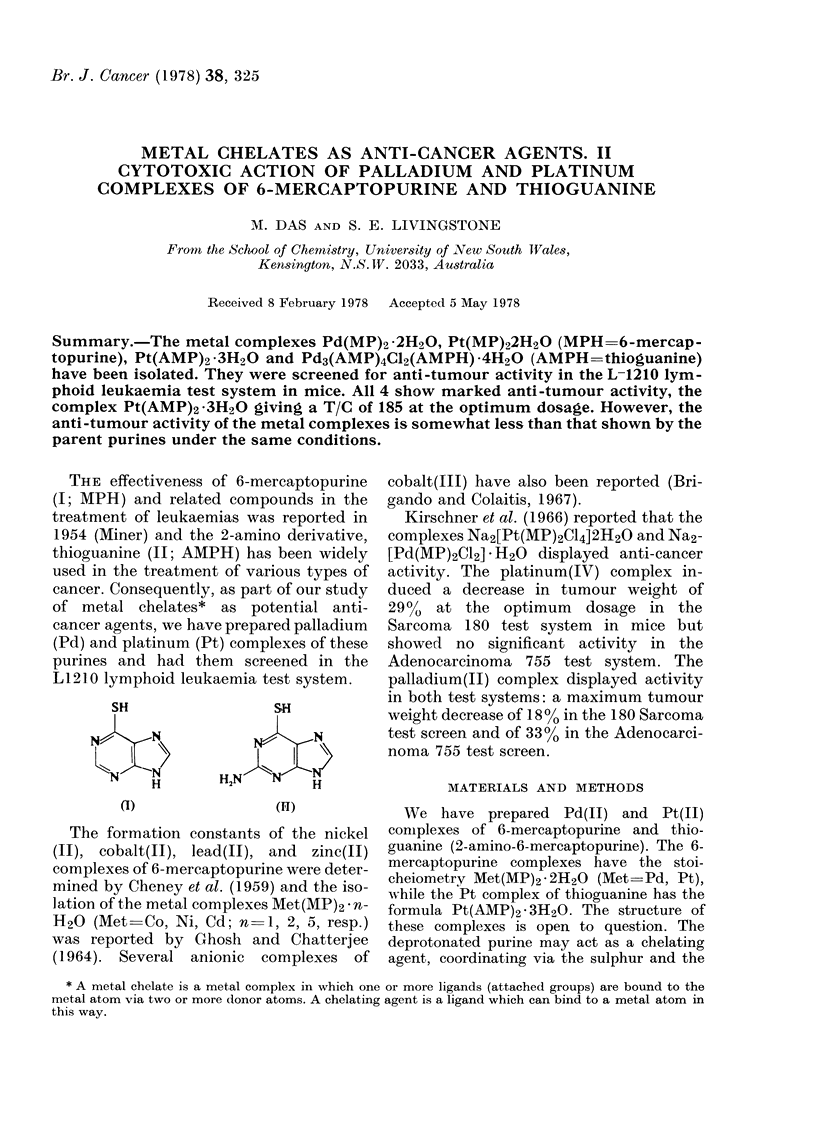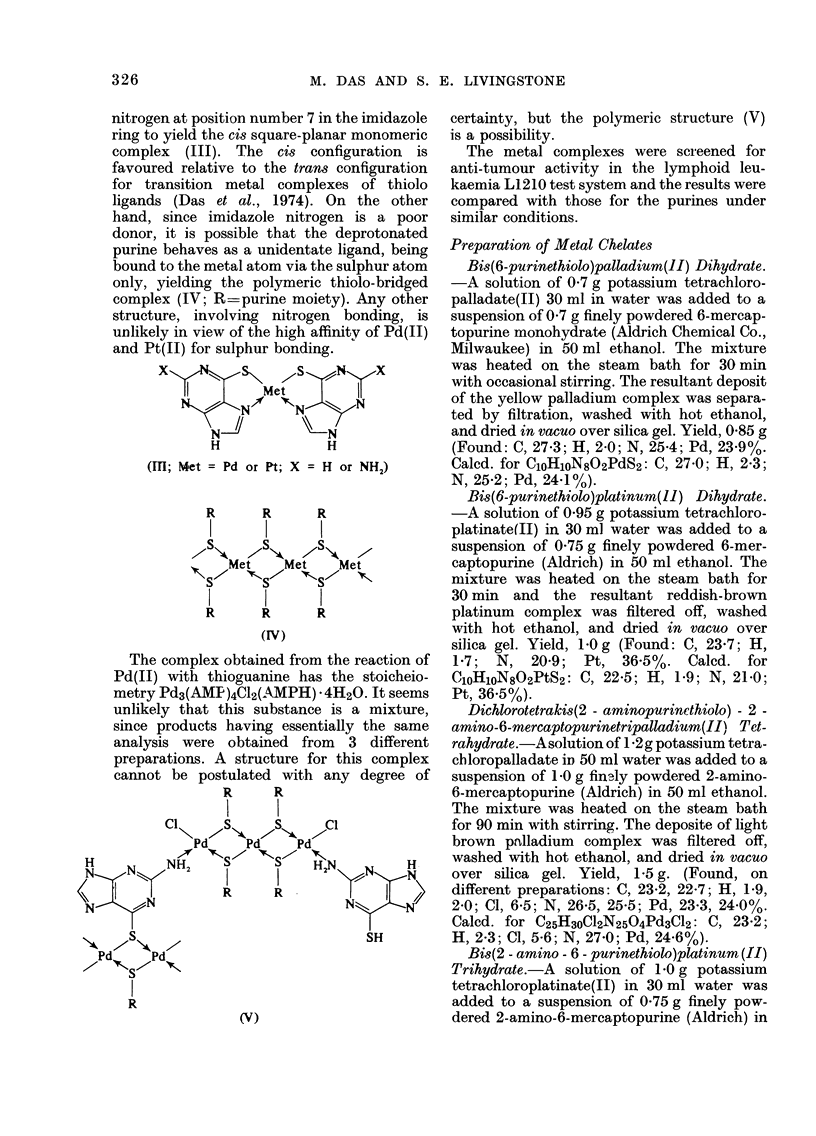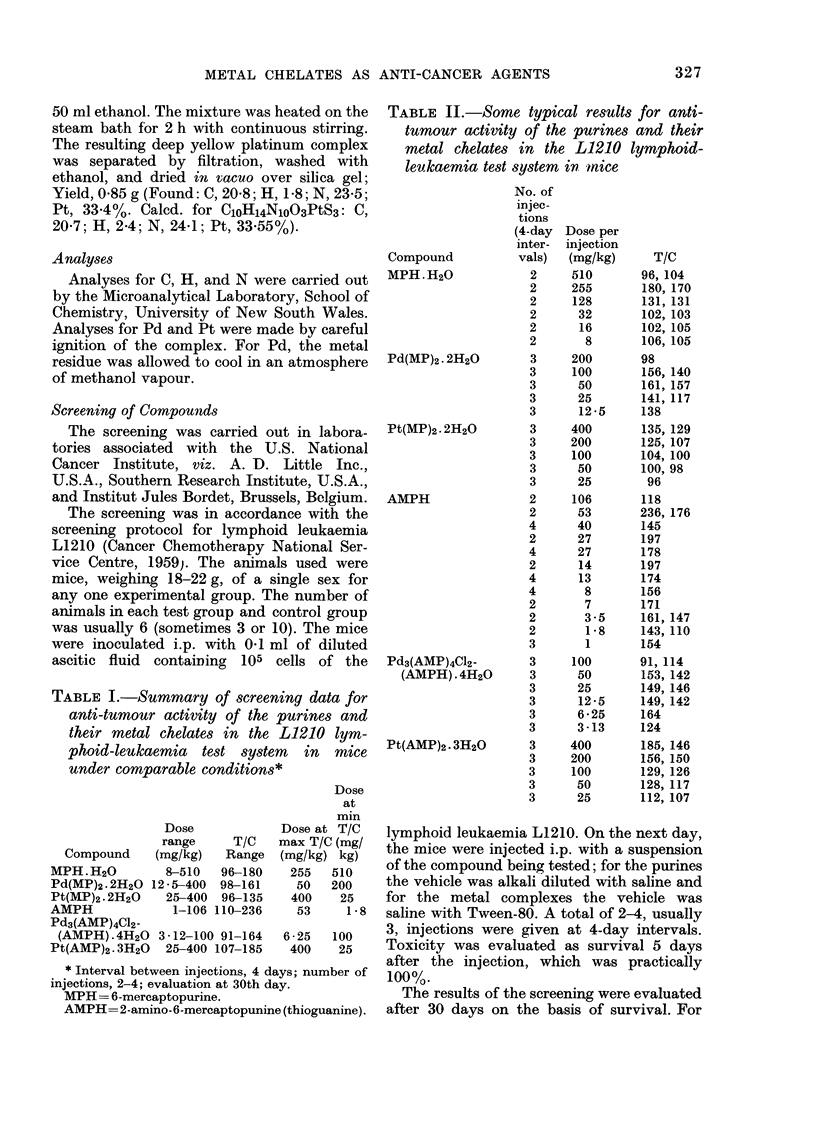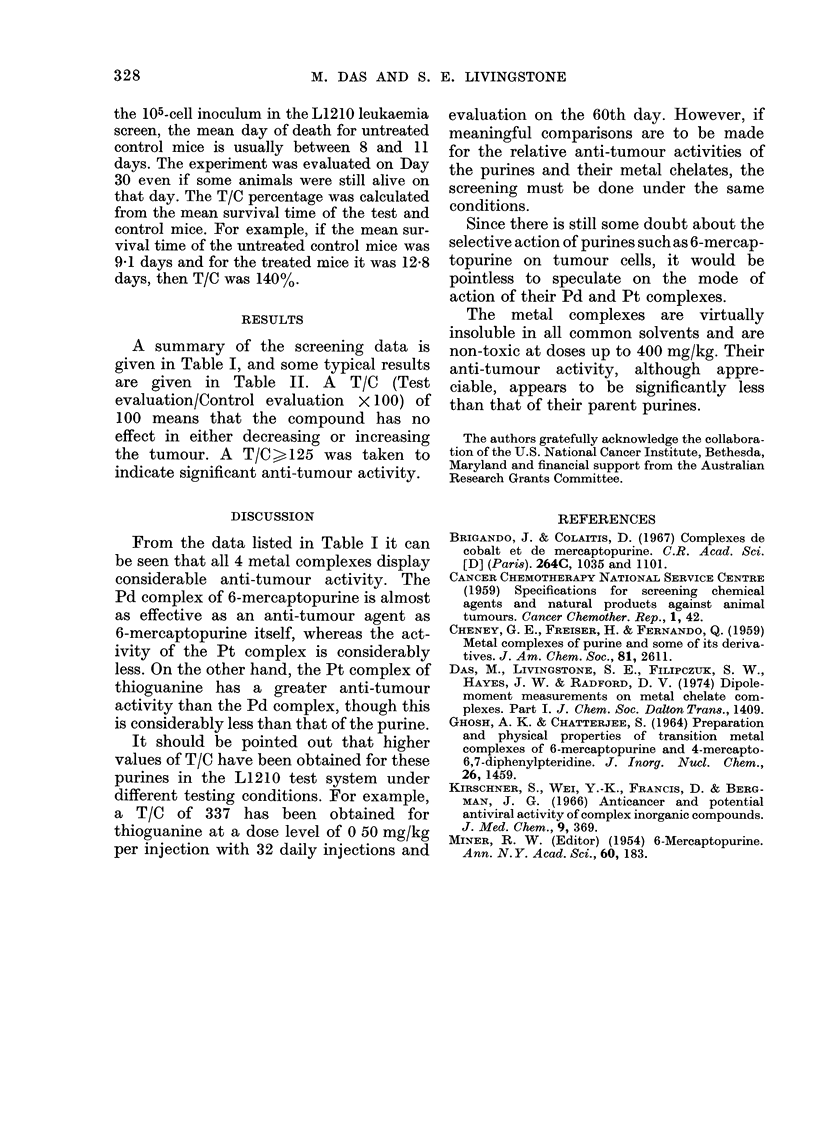# Metal chelates as anti-cancer agents. II cytotoxic action of palladium and platinum complexes of 6-mercaptopurine and thioguanine.

**DOI:** 10.1038/bjc.1978.205

**Published:** 1978-08

**Authors:** M. Das, S. E. Livingstone

## Abstract

The metal complexes Pd(MP)2.2H2O, Pt(MP)2H2O (MPH=6-mercaptopurine), Pt(AMP2.3H2O and Pd3(AMP)4Cl2(AMPH).4H2O (AMPH=thioguanine) have been isolated. They were screened for anti-tumour activity in the L-1210 lymphoid leukaemia test system in mice. All 4 show marked anti-tumour activity, the complex Pt(AMP)2.3H2O giving a T/C of 185 at the optimum dosage. However, the anti-tumour activity of the metal complexes is somewhat less than that shown by the parent purines under the same conditions.


*Br. J. Cancer* (1978) **38**, 325

### METAL CHELATES AS ANTI-CANCER AGENTS. II CYTOTOXIC ACTION OF PALLADIUM AND PLATINUM COMPLEXES OF 6-MERCAPTOPURINE AND THIOGUANINE

M. DAS AND S. E. LIVINGSTONE

*From the School of Chemistry, University of New South Wales, Kensington, N.S.W. 2033, Australia*

Received 8 February 1978 Accepted 5 May 1978

**Summary.—The metal complexes $Pd(MP)_2 \cdot 2H_2O$, $Pt(MP)_2 2H_2O$ (MPH=6-mercaptopurine), $Pt(AMP)_2 \cdot 3H_2O$ and $Pd_3(AMP)_4Cl_2(AMPH) \cdot 4H_2O$ (AMPH=thioguanine) have been isolated. They were screened for anti-tumour activity in the L-1210 lymphoid leukaemia test system in mice. All 4 show marked anti-tumour activity, the complex $Pt(AMP)_2 \cdot 3H_2O$ giving a T/C of 185 at the optimum dosage. However, the anti-tumour activity of the metal complexes is somewhat less than that shown by the parent purines under the same conditions.**

THE effectiveness of 6-mercaptopurine (I; MPH) and related compounds in the treatment of leukaemias was reported in 1954 (Miner) and the 2-amino derivative, thioguanine (II; AMPH) has been widely used in the treatment of various types of cancer. Consequently, as part of our study of metal chelates* as potential anti-cancer agents, we have prepared palladium (Pd) and platinum (Pt) complexes of these purines and had them screened in the L1210 lymphoid leukaemia test system.

(I)

(II)

The formation constants of the nickel (II), cobalt(II), lead(II), and zinc(II) complexes of 6-mercaptopurine were determined by Cheney *et al.* (1959) and the isolation of the metal complexes $Met(MP)_2 \cdot n$-$H_2O$ (Met=Co, Ni, Cd; $n$=1, 2, 5, resp.) was reported by Ghosh and Chatterjee (1964). Several anionic complexes of cobalt(III) have also been reported (Brigando and Colaitis, 1967).

Kirschner *et al.* (1966) reported that the complexes $Na_2[Pt(MP)_2Cl_4]2H_2O$ and $Na_2$-$[Pd(MP)_2Cl_2] \cdot H_2O$ displayed anti-cancer activity. The platinum(IV) complex induced a decrease in tumour weight of 29% at the optimum dosage in the Sarcoma 180 test system in mice but showed no significant activity in the Adenocarcinoma 755 test system. The palladium(II) complex displayed activity in both test systems: a maximum tumour weight decrease of 18% in the 180 Sarcoma test screen and of 33% in the Adenocarcinoma 755 test screen.

### MATERIALS AND METHODS

We have prepared Pd(II) and Pt(II) complexes of 6-mercaptopurine and thioguanine (2-amino-6-mercaptopurine). The 6-mercaptopurine complexes have the stoicheiometry $Met(MP)_2 \cdot 2H_2O$ (Met=Pd, Pt), while the Pt complex of thioguanine has the formula $Pt(AMP)_2 \cdot 3H_2O$. The structure of these complexes is open to question. The deprotonated purine may act as a chelating agent, coordinating via the sulphur and the

* A metal chelate is a metal complex in which one or more ligands (attached groups) are bound to the metal atom via two or more donor atoms. A chelating agent is a ligand which can bind to a metal atom in this way.

nitrogen at position number 7 in the imidazole ring to yield the *cis* square-planar monomeric complex (III). The *cis* configuration is favoured relative to the *trans* configuration for transition metal complexes of thiolo ligands (Das *et al.*, 1974). On the other hand, since imidazole nitrogen is a poor donor, it is possible that the deprotonated purine behaves as a unidentate ligand, being bound to the metal atom via the sulphur atom only, yielding the polymeric thiolo-bridged complex (IV; R=purine moiety). Any other structure, involving nitrogen bonding, is unlikely in view of the high affinity of Pd(II) and Pt(II) for sulphur bonding.

(III; Met = Pd or Pt; X = H or $NH_2$)

(IV)

The complex obtained from the reaction of Pd(II) with thioguanine has the stoicheiometry $Pd_3(AMP)_4Cl_2(AMPH)\cdot 4H_2O$. It seems unlikely that this substance is a mixture, since products having essentially the same analysis were obtained from 3 different preparations. A structure for this complex cannot be postulated with any degree of certainty, but the polymeric structure (V) is a possibility.

(V)

The metal complexes were screened for anti-tumour activity in the lymphoid leukaemia L1210 test system and the results were compared with those for the purines under similar conditions.

*Preparation of Metal Chelates*

*Bis(6-purinethiolo)palladium(II) Dihydrate.*—A solution of 0·7 g potassium tetrachloropalladate(II) 30 ml in water was added to a suspension of 0·7 g finely powdered 6-mercaptopurine monohydrate (Aldrich Chemical Co., Milwaukee) in 50 ml ethanol. The mixture was heated on the steam bath for 30 min with occasional stirring. The resultant deposit of the yellow palladium complex was separated by filtration, washed with hot ethanol, and dried *in vacuo* over silica gel. Yield, 0·85 g (Found: C, 27·3; H, 2·0; N, 25·4; Pd, 23·9%. Calcd. for $C_{10}H_{10}N_8O_2PdS_2$: C, 27·0; H, 2·3; N, 25·2; Pd, 24·1%).

*Bis(6-purinethiolo)platinum(II) Dihydrate.*—A solution of 0·95 g potassium tetrachloroplatinate(II) in 30 ml water was added to a suspension of 0·75 g finely powdered 6-mercaptopurine (Aldrich) in 50 ml ethanol. The mixture was heated on the steam bath for 30 min and the resultant reddish-brown platinum complex was filtered off, washed with hot ethanol, and dried *in vacuo* over silica gel. Yield, 1·0 g (Found: C, 23·7; H, 1·7; N, 20·9; Pt, 36·5%. Calcd. for $C_{10}H_{10}N_8O_2PtS_2$: C, 22·5; H, 1·9; N, 21·0; Pt, 36·5%).

*Dichlorotetrakis(2 - aminopurinethiolo) - 2 - amino-6-mercaptopurinetripalladium(II) Tetrahydrate.*—A solution of 1·2 g potassium tetrachloropalladate in 50 ml water was added to a suspension of 1·0 g finely powdered 2-amino-6-mercaptopurine (Aldrich) in 50 ml ethanol. The mixture was heated on the steam bath for 90 min with stirring. The deposite of light brown palladium complex was filtered off, washed with hot ethanol, and dried *in vacuo* over silica gel. Yield, 1·5 g. (Found, on different preparations: C, 23·2, 22·7; H, 1·9, 2·0; Cl, 6·5; N, 26·5, 25·5; Pd, 23·3, 24·0%. Calcd. for $C_{25}H_{30}Cl_2N_{25}O_4Pd_3Cl_2$: C, 23·2; H, 2·3; Cl, 5·6; N, 27·0; Pd, 24·6%).

*Bis(2 - amino - 6 - purinethiolo)platinum (II) Trihydrate.*—A solution of 1·0 g potassium tetrachloroplatinate(II) in 30 ml water was added to a suspension of 0·75 g finely powdered 2-amino-6-mercaptopurine (Aldrich) in

50 ml ethanol. The mixture was heated on the steam bath for 2 h with continuous stirring. The resulting deep yellow platinum complex was separated by filtration, washed with ethanol, and dried *in vacuo* over silica gel; Yield, 0·85 g (Found: C, 20·8; H, 1·8; N, 23·5; Pt, 33·4%. Calcd. for $C_{10}H_{14}N_{10}O_3PtS_3$: C, 20·7; H, 2·4; N, 24·1; Pt, 33·55%).

### *Analyses*

Analyses for C, H, and N were carried out by the Microanalytical Laboratory, School of Chemistry, University of New South Wales. Analyses for Pd and Pt were made by careful ignition of the complex. For Pd, the metal residue was allowed to cool in an atmosphere of methanol vapour.

### *Screening of Compounds*

The screening was carried out in laboratories associated with the U.S. National Cancer Institute, *viz.* A. D. Little Inc., U.S.A., Southern Research Institute, U.S.A., and Institut Jules Bordet, Brussels, Belgium.

The screening was in accordance with the screening protocol for lymphoid leukaemia L1210 (Cancer Chemotherapy National Service Centre, 1959). The animals used were mice, weighing 18–22 g, of a single sex for any one experimental group. The number of animals in each test group and control group was usually 6 (sometimes 3 or 10). The mice were inoculated i.p. with 0·1 ml of diluted ascitic fluid containing $10^5$ cells of the lymphoid leukaemia L1210. On the next day, the mice were injected i.p. with a suspension of the compound being tested; for the purines the vehicle was alkali diluted with saline and for the metal complexes the vehicle was saline with Tween-80. A total of 2–4, usually 3, injections were given at 4-day intervals. Toxicity was evaluated as survival 5 days after the injection, which was practically 100%.

The results of the screening were evaluated after 30 days on the basis of survival. For

TABLE I.—*Summary of screening data for anti-tumour activity of the purines and their metal chelates in the L1210 lymphoid-leukaemia test system in mice under comparable conditions**

| Compound | Dose range (mg/kg) | T/C Range | Dose at max T/C (mg/kg) | Dose at min T/C (mg/kg) |
|---|---|---|---|---|
| $MPH.H_2O$ | 8–510 | 96–180 | 255 | 510 |
| $Pd(MP)_2.2H_2O$ | 12·5–400 | 98–161 | 50 | 200 |
| $Pt(MP)_2.2H_2O$ | 25–400 | 96–135 | 400 | 25 |
| AMPH | 1–106 | 110–236 | 53 | 1·8 |
| $Pd_3(AMP)_4Cl_2$-$(AMPH).4H_2O$ | 3·12–100 | 91–164 | 6·25 | 100 |
| $Pt(AMP)_2.3H_2O$ | 25–400 | 107–185 | 400 | 25 |

* Interval between injections, 4 days; number of injections, 2–4; evaluation at 30th day.
MPH = 6-mercaptopurine.
AMPH = 2-amino-6-mercaptopunine (thioguanine).

TABLE II.—*Some typical results for anti-tumour activity of the purines and their metal chelates in the L1210 lymphoid-leukaemia test system in mice*

| Compound | No. of injections (4-day intervals) | Dose per injection (mg/kg) | T/C |
|---|---|---|---|
| $MPH.H_2O$ | 2 | 510 | 96, 104 |
| | 2 | 255 | 180, 170 |
| | 2 | 128 | 131, 131 |
| | 2 | 32 | 102, 103 |
| | 2 | 16 | 102, 105 |
| | 2 | 8 | 106, 105 |
| $Pd(MP)_2.2H_2O$ | 3 | 200 | 98 |
| | 3 | 100 | 156, 140 |
| | 3 | 50 | 161, 157 |
| | 3 | 25 | 141, 117 |
| | 3 | 12·5 | 138 |
| $Pt(MP)_2.2H_2O$ | 3 | 400 | 135, 129 |
| | 3 | 200 | 125, 107 |
| | 3 | 100 | 104, 100 |
| | 3 | 50 | 100, 98 |
| | 3 | 25 | 96 |
| AMPH | 2 | 106 | 118 |
| | 2 | 53 | 236, 176 |
| | 4 | 40 | 145 |
| | 2 | 27 | 197 |
| | 4 | 27 | 178 |
| | 2 | 14 | 197 |
| | 4 | 13 | 174 |
| | 4 | 8 | 156 |
| | 2 | 7 | 171 |
| | 2 | 3·5 | 161, 147 |
| | 2 | 1·8 | 143, 110 |
| | 3 | 1 | 154 |
| $Pd_3(AMP)_4Cl_2$-$(AMPH).4H_2O$ | 3 | 100 | 91, 114 |
| | 3 | 50 | 153, 142 |
| | 3 | 25 | 149, 146 |
| | 3 | 12·5 | 149, 142 |
| | 3 | 6·25 | 164 |
| | 3 | 3·13 | 124 |
| $Pt(AMP)_2.3H_2O$ | 3 | 400 | 185, 146 |
| | 3 | 200 | 156, 150 |
| | 3 | 100 | 129, 126 |
| | 3 | 50 | 128, 117 |
| | 3 | 25 | 112, 107 |

the $10^5$-cell inoculum in the L1210 leukaemia screen, the mean day of death for untreated control mice is usually between 8 and 11 days. The experiment was evaluated on Day 30 even if some animals were still alive on that day. The T/C percentage was calculated from the mean survival time of the test and control mice. For example, if the mean survival time of the untreated control mice was 9·1 days and for the treated mice it was 12·8 days, then T/C was 140%.

### RESULTS

A summary of the screening data is given in Table I, and some typical results are given in Table II. A T/C (Test evaluation/Control evaluation $\times 100$) of 100 means that the compound has no effect in either decreasing or increasing the tumour. A T/C$\geqslant$125 was taken to indicate significant anti-tumour activity.

### DISCUSSION

From the data listed in Table I it can be seen that all 4 metal complexes display considerable anti-tumour activity. The Pd complex of 6-mercaptopurine is almost as effective as an anti-tumour agent as 6-mercaptopurine itself, whereas the activity of the Pt complex is considerably less. On the other hand, the Pt complex of thioguanine has a greater anti-tumour activity than the Pd complex, though this is considerably less than that of the purine.

It should be pointed out that higher values of T/C have been obtained for these purines in the L1210 test system under different testing conditions. For example, a T/C of 337 has been obtained for thioguanine at a dose level of 0 50 mg/kg per injection with 32 daily injections and evaluation on the 60th day. However, if meaningful comparisons are to be made for the relative anti-tumour activities of the purines and their metal chelates, the screening must be done under the same conditions.

Since there is still some doubt about the selective action of purines such as 6-mercaptopurine on tumour cells, it would be pointless to speculate on the mode of action of their Pd and Pt complexes.

The metal complexes are virtually insoluble in all common solvents and are non-toxic at doses up to 400 mg/kg. Their anti-tumour activity, although appreciable, appears to be significantly less than that of their parent purines.

The authors gratefully acknowledge the collaboration of the U.S. National Cancer Institute, Bethesda, Maryland and financial support from the Australian Research Grants Committee.

### REFERENCES

BRIGANDO, J. & COLAITIS, D. (1967) Complexes de cobalt et de mercaptopurine. *C.R. Acad. Sci.* [D] (*Paris*). **264C**, 1035 and 1101.

CANCER CHEMOTHERAPY NATIONAL SERVICE CENTRE (1959) Specifications for screening chemical agents and natural products against animal tumours. *Cancer Chemother. Rep.*, **1**, 42.

CHENEY, G. E., FREISER, H. & FERNANDO, Q. (1959) Metal complexes of purine and some of its derivatives. *J. Am. Chem. Soc.*, **81**, 2611.

DAS, M., LIVINGSTONE, S. E., FILIPCZUK, S. W., HAYES, J. W. & RADFORD, D. V. (1974) Dipole-moment measurements on metal chelate complexes. Part I. *J. Chem. Soc. Dalton Trans.*, 1409.

GHOSH, A. K. & CHATTERJEE, S. (1964) Preparation and physical properties of transition metal complexes of 6-mercaptopurine and 4-mercapto-6,7-diphenylpteridine. *J. Inorg. Nucl. Chem.*, **26**, 1459.

KIRSCHNER, S., WEI, Y.-K., FRANCIS, D. & BERGMAN, J. G. (1966) Anticancer and potential antiviral activity of complex inorganic compounds. *J. Med. Chem.*, **9**, 369.

MINER, R. W. (Editor) (1954) 6-Mercaptopurine. *Ann. N.Y. Acad. Sci.*, **60**, 183.